# Toxin-mediated ribosome stalling reprograms the *Mycobacterium tuberculosis* proteome

**DOI:** 10.1038/s41467-019-10869-8

**Published:** 2019-07-10

**Authors:** Valdir C. Barth, Ju-Mei Zeng, Irina O. Vvedenskaya, Ming Ouyang, Robert N. Husson, Nancy A. Woychik

**Affiliations:** 1 Department of Biochemistry and Molecular Biology, Rutgers University, Robert Wood Johnson Medical School, Piscataway, NJ 08854 USA; 2 Division of Infectious Diseases, Boston Children’s Hospital and Harvard Medical School, Boston, MA 02115 USA; 3 Waksman Institute, Rutgers University, Piscataway, NJ 08854 USA; 4 Department of Genetics, Rutgers University, Piscataway, NJ 08854 USA; 5 University of Massachusetts-Boston, 100 William T Morrissey Blvd, Boston, MA 02125 USA; 6 Rutgers Cancer Institute of New Jersey, New Brunswick, NJ 08901 USA

**Keywords:** Bacterial toxins, Pathogens, Ribosome, tRNAs

## Abstract

*Mycobacterium tuberculosis* readily adapts to survive a wide range of assaults by modifying its physiology and establishing a latent tuberculosis (TB) infection. Here we report a sophisticated mode of regulation by a tRNA-cleaving toxin that enlists highly selective ribosome stalling to recalibrate the transcriptome and remodel the proteome. This toxin, MazF-mt9, exclusively inactivates one isoacceptor tRNA, tRNA^Lys43-UUU^, through cleavage at a single site within its anticodon (UU↓U). Because wobble rules preclude compensation for loss of tRNA^Lys43-UUU^ by the second *M. tuberculosis* lysine tRNA, tRNA^Lys19-CUU^, ribosome stalling occurs at in-frame cognate AAA Lys codons. Consequently, the transcripts harboring these stalled ribosomes are selectively cleaved by specific RNases, leading to their preferential deletion. This surgically altered transcriptome generates concomitant changes to the proteome, skewing synthesis of newly synthesized proteins away from those rich in AAA Lys codons toward those harboring few or no AAA codons. This toxin-mediated proteome reprogramming may work in tandem with other pathways to facilitate *M. tuberculosis* stress survival.

## Abstract (web summary)

MazF endoribonucleases are thought to arrest growth of *Mycobacterium tuberculosis* by global translation inhibition. Here, Barth et al. show that MazF-mt9 cleaves a specific tRNA, leading to ribosome stalling at AAA codons and thus selective mRNA degradation and changes in transcriptome and proteome.

## Introduction

The molecular switches that enable *M. tuberculosis* to slow or stop replication, become dormant and establish latent tuberculosis infection are poorly characterized. Toxin–antitoxin (TA) systems/modules are thought to be involved in *M. tuberculosis* stress survival and the establishment of latent tuberculosis infection because they typically impart reversible growth inhibition in their host in response to stresses relevant to this state^1^. TA systems are operons comprising adjacent genes encoding two small (~10 kDa) proteins, a toxin and its cognate antitoxin that inhibits toxin activity through formation of a stable TA protein–protein complex. Stress conditions lead to lower levels of the antitoxin and thus, a preponderance of free toxin which exerts it growth-regulating and/or other functions from within the bacterial cells^1^. In fact, *M. tuberculosis* cells subjected to stresses relevant to latent tuberculosis infection—nutrient limitation^2–5^, hypoxia^5–7^, macrophage infection^6,8–10^, or antibiotic treatment^5,11–13^—exhibit enhanced expression of TA toxins. Thus, the phenotypes associated with toxin expression in *M. tuberculosis* are consistent with a role for TA systems in the establishment and maintenance of latent tuberculosis and persistence of this pathogen.

Among the approximately 90 TA systems in *M. tuberculosis*, 11 belong to the MazEF family^6,14^. All MazF toxins are unified by their hallmark sequence-specific endoribonuclease activity. With a single exception, all MazF toxins in bacteria cleave mRNA and/or rRNA at specific three-base, five-base, or seven-base recognition sequences^1^. The exception, the *M. tuberculosis* MazF-mt9 toxin (aka MazF7, Rv2063A), specifically recognizes and cleaves tRNA based on both sequence and structure determinants^15,16^. However, the series of downstream events that lead to growth arrest following MazF toxin-mediated cleavage of the *M. tuberculosis* target RNA are not well understood, especially for tRNA-cleaving toxins.

It has been widely assumed that since these toxins cleave one or more RNAs involved in protein synthesis—mRNA, rRNA, and/or tRNA—they arrest growth by global translation inhibition^1,17–19^. However, deployment of 88 toxins to reach the same endpoint represents a redundancy that is at odds with the relatively compact *M. tuberculosis* genome adapted for survival within host granulomas during latent tuberculosis. Here we report the molecular mechanism of toxin MazF-mt9, which demonstrates that tRNA-cleaving *M. tuberculosis* toxins do not necessarily act by simply inhibiting translation, it illuminates a sophisticated mode of transcriptome recalibration and proteome reprogramming through highly selective ribosome stalling.

## Results

### MazF-mt9 inactivates a single tRNA in *M. tuberculosis*

In our earlier work, tRNA was identified as the primary target of MazF-mt9—the first MazF toxin to exhibit a preference for tRNA—but we had not expressed this toxin in *M. tuberculosis* cells to identify the true target in vivo. Here we enlisted our specialized RNA-seq method^20^, 5′ RNA-seq, to specifically identify the RNA(s) cleaved by the MazF-mt9 toxin in *M. tuberculosis* cells, as well as the precise site of cleavage within the RNA(s). 5′ RNA-seq differentially detects one or more subpopulations of RNA depending on the modification present at the 5′ end of the transcript. The 5′ RNA-seq method used here selectively detected transcripts with a 5′-hydroxyl (OH) moiety generated by MazF-mt9 and other MazF family toxins^21^.

**Fig. 1:**
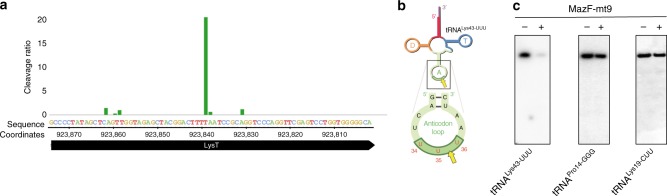
MazF-mt9 targets only tRNA^Lys43-UUU^ in vivo. **a** Histogram representing the ratio of cleavage by MazF-mt9 identified using 5′ RNA-seq at each nucleotide within the *lysT* gene (tRNA^Lys43-UUU^) in *M. tuberculosis* H37Rv after 7 days of toxin induction. Genomic positions and the negative strand sequence are shown. **b** Representation of the only MazF-mt9 target, tRNA^Lys43-UUU^ (adapted from Schifano et al.^15^). Anticodon (UUU), in red; cleavage site, yellow arrow. **c** Northern analysis of RNA from ±MazF-mt9 *M. tuberculosis* cells using the tRNA isoacceptor-specific oligonucleotides indicated. Uncropped Northern blot images are provided as a tab within the Source Data file

Only one tRNA, the Lys tRNA^Lys43-UUU^ isoacceptor, was identified as the primary target of MazF-mt9 when 5′ RNA-seq was performed on *M. tuberculosis* H37Rv cells expressing MazF-mt9 versus control cells (Fig. 1a). None of the other 44 *M. tuberculosis* tRNA species were cleaved by MazF-mt9, not even the other isoacceptor Lys tRNA, tRNA^Lys19-CUU^ (Supplementary Fig. 1a,c). Cleavage occurred before the third U, i.e., ^35^UU↓U^37^, within the anticodon sequence (Fig. 1b). This contrasted with our earlier 5′ RNA-seq of *M. tuberculosis* RNA incubated with recombinant MazF-mt9^15^. In that in vitro experiment, both tRNA^Lys43-UUU^ and tRNA^Pro14-GGG^ were targeted by MazF-mt9. Both tRNAs contain a UUU consensus sequence, tRNA^Lys43-UUU^ within its single-stranded anticodon-loop or tRNA^Pro14-GGG^ in its D-loop^15^. Many UUU-containing transcripts were also cleaved in vitro when the secondary structure of the UU↓U cleavage site and flanking sequences happen to mimic that of a tRNA anticodon stem loop^15^.

Since three tRNA targets were previously identified in vitro, we used northern analysis to validate our in vivo 5′ RNA-seq result, which identified tRNA^Lys43-UUU^ as the sole tRNA target (Fig. 1c). Consistent with our in vivo 5′ RNA-seq data, only tRNA^Lys43-UUU^ was cleaved upon expression of MazF-mt9 in *M. tuberculosis* cells. Therefore, the MazF-mt9 toxin needs to be expressed in living cells, not simply incubated with *M. tuberculosis* RNA in vitro, to accurately pinpoint the sole tRNA target. This requirement for in vivo expression in the natural host is likely broadly relevant for accurate target identification for the other *M. tuberculosis* RNA-cleaving toxins.

### 5′ RNA-seq suggests ribosome stalling at AAA Lys codons

**Fig. 2:**
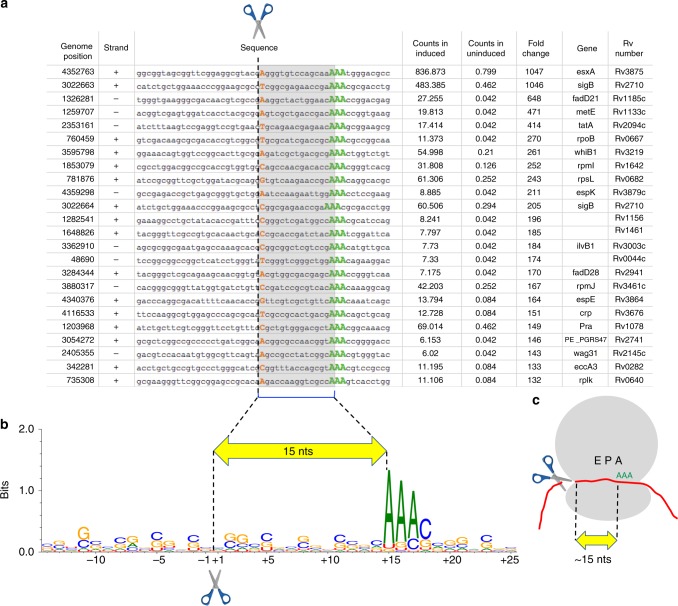
5′ RNA-seq captures ribosome stalling events at AAA Lys codons. **a** Top mRNA hits detected by 5′ RNA seq, ±25 nts surrounding the cleavage site (scissor). The first nucleotide of each mapped read, orange; AAA Lys codons, green. Counts are represented as reads per million (rpm). **b** Nucleotide frequency logo (weblogo, see ref. ^37^) at each position constructed from the top 50 of the 181 mRNA hits identified. Positions numbered relative to the cleavage site. **c** Schematic of a ribosome (gray) and the ~15 nt length (yellow arrow) from the 5′ end of transcript to the A-site. Source data are provided in Supplementary Data 1

In addition to identification of tRNA^Lys43-UUU^ as the primary target of MazF-mt9, three unique features were uncovered in the 5′ RNA-seq dataset from *M. tuberculosis* H37Rv that suggested ribosome stalling was intrinsic to the mechanism of action of MazF-mt9. First, in addition to lysine tRNA^Lys43-UUU^, there were nearly two hundred mRNAs that were also significantly cleaved (fold change >10) when MazF-mt9 was expressed (Supplementary Data 1). Second, there was a conspicuous fixed distance, ~15 nt, between the cleavage site in these mRNAs and the presence of an in-frame AAA Lys codon Fig. 2a, b). Third, there was no consensus sequence at the cut site (Fig. 2a, b), indicating that these mRNAs were not cleaved by MazF-mt9 (which only cleaves UU↓U sequences in the proper structural context^15^). Instead, these mRNAs were cut by another RNase, whose activity was detectable because it also generated an RNA cleavage product with a 5′-OH.

We first investigated the significance of the 15 nt periodicity in the 5′ RNA-seq dataset. The precise distance between the 5′-OH cleavage sites and the AAA Lys codons (~15 nts) suggested that ribosomes were stalling at the AAA Lys codon because its cognate tRNA^Lys43-UUU^ was depleted. It is known that the bacterial ribosome footprint is ~28 nt^22^ and the distance from the P-site to the 3′ end of this footprint is 15 nts^22^. Therefore, we posited that the ~15 nt gap between the 5′ RNA-seq cleavage site and the in-frame AAA Lys codon represented the ribosome footprint from the 5′ end to the tRNA^Lys43-UUU^ codon at the A-site (illustrated in Fig. 2c).

### Ribo-seq confirms ribosome stalling

**Fig. 3:**
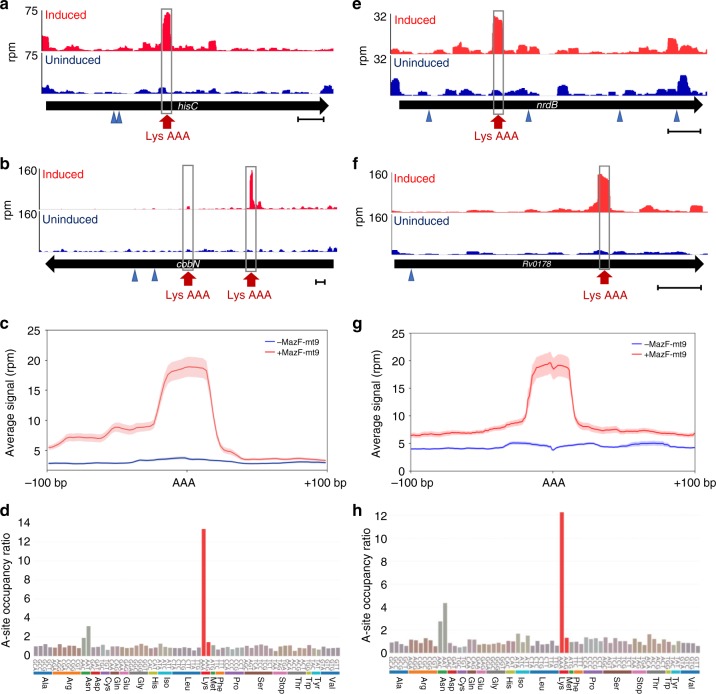
Ribo-seq confirms transcriptome-wide AAA Lys-specific ribosome stalling. **a**, **b**, **e**, **f** Ribo-seq read coverage in reads per million (rpm) of AAA Lys-containing *M. smegmatis* (**a**, **b**) or *M. tuberculosis* mc^2^ 6206 (**e**, **f**) transcripts in ±MazF-mt9 cells (arrows indicate direction of translation). The location of in-frame AAA Lys codons and out-of-frame AAAs are indicated (red arrow/gray box and blue arrow head, respectively). Scale bars illustrate a 100 bp genomic distance. **c**, **g**, Average read counts (rpm) of mapped ribosomes surrounding AAA Lys codons in +MazF-mt9 (red line) or -MazF-mt9 (blue) cells; light shading represents the standard deviation. **d**, **h** Ratio of codon occupancy (MazF-mt9 induced vs. uninduced) at the predicted ribosomal A-site position identified in the mapped ribosomal footprints, grouped by amino acid or stop codon. Source data for **d** and **h** are provided in a separate tab within the Source Data file

**Fig. 4:**
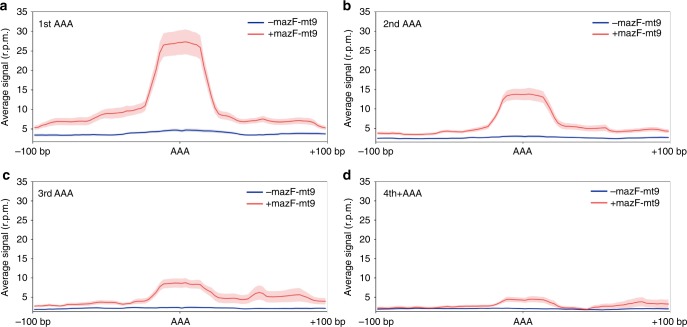
Ribo-seq shows ribosome stalling preference for early AAA codons. **a**–**d** Average read counts (reads per million, rpm) of mapped ribosome footprints surrounding the first (**a**), second (**b**), third (**c**), or fourth and higher (**d**) AAA Lys codon of *M. smegmatis* genes. Blue or red solid line represents uninduced cells or cells expressing MazF-mt9, respectively, with the light-colored shading denoting standard deviation

To confirm that the ~15 nt gap between the cleavage site and the in-frame AAA Lys represented ribosome stalling at AAA codons, we performed Ribo-seq of MazF-mt9-expressing *M. smegmatis* and *M. tuberculosis* cells (Fig. 3). MazF-mt9 cleaves tRNA^Lys23-UUU^ and exhibits the same telltale markers of stalling as *M. tuberculosis* when analyzed by 5′ RNA-seq. Four representative transcripts, two from *M. smegmatis* (*hisC* and *cobN*, Fig. 3a, b) and two from *M. tuberculosis* (*nrdB* and *Rv0178*, Fig. 3e, f) revealed exclusive stalling at in-frame Lys AAA codons. There was no ribosome stalling at out-of-frame AAA codons (blue arrows, Fig. 3a, b, e, f). In fact, this striking preference for ribosome stalling at AAA Lys codons was consistent throughout the transcriptomes of each mycobacteria species when stalling was analyzed at AAA codons only (Fig. 3c, g) or when codon occupancy for mapped ribosomal footprints of all codons was compared (Fig. 3d, h). Not only was there a preference for AAA Lys codons, stalling occurred predominantly at the first AAA Lys codon in any given mRNA, as the number of stalled ribosomes captured at the second, third or fourth AAA Lys codon by Ribo-seq successively diminished (Fig. 4). Consistent with the northern analysis in Fig. 1c, we did not observe ribosome stalling at tRNA^Pro14-GGG^ (that we previously reported was cleaved by MazF-mt9 in vitro^15^) or at the other Lys isoacceptor tRNA^Lys19-CUU^ (Supplementary Fig. 1b, c).

### Ribosome stalling at AAAs upon RNase J expression in *E. coli*

Next we sought to identify the RNase that cut on the 5′ side of the stalled ribosome and generated a 5′-OH detectable by 5′ RNA-seq. Although typically involved in rRNA maturation and mRNA turnover^23^, we suspected RNase J because it has been reported to cleave on the 5′ side of erythromycin-stalled ribosomes in *Bacillus subtilis* ~15 nt from the A-site^24^. Therefore, *M. tuberculosis* RNase J (Rv2752c) was ectopically expressed in *E. coli* cells to determine if ribosome stalling was now detectable. We chose *E. coli* because it naturally lacks this enzyme and has no other known RNase J-like activity. Also, MazF-mt9 expression in *E. coli* has two essential features that enable us to assay for RNase J gain-of-function activity. First, as in *M. tuberculosis*, the only tRNA that MazF-mt9 cleaves in *E. coli* is tRNA^LysUUU^ ^15^. Second, even though tRNA^LysUUU^ was exclusively cleaved, there was no evidence of ribosome stalling in our 5′ RNA-seq dataset^15^.

**Fig. 5:**
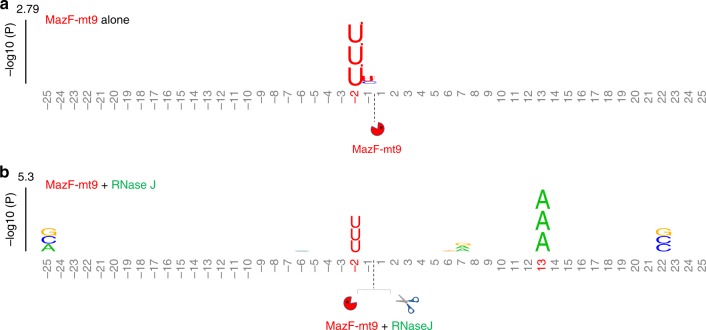
*M. tuberculosis* RNase J cleaves 5′ of stalled ribosomes favoring expression of AAA-deficient transcripts. KpLogo^38^ graphs derived from *E. coli* 5′ RNA-seq + MazF-mt9 (**a**) or +MazF-mt9 + RNase J (**b**) datasets compared to -MazF-mt9 samples. The enriched k-mers in the datasets are stacked vertically at the position of their initial nucleotide. The positions are numbered relative to the cleavage site (dotted line), with those achieving statistical significance (Bonferroni corrected *p*-value < 0.01) shown in red

The kplogo in Fig. 5 illustrates the key features of the experimental design and outcome. As is the case with incubation of recombinant MazF-mt9 with total RNA from *M. tuberculosis*, MazF-mt9 expression in *E. coli* does not exhibit exclusive specificity for tRNA^LysUUU^, it also cleaves some mRNAs at UU↓U that are predicted to form structures that recapitulate the tRNA^LysUUU^ anticodon stem loop^15^ (reflected as a stacked UUU at −2 using kplogo in Fig. 5a). However, the presence of these cleaved mRNAs did not interfere with interpretation of this experiment because we looked for a phenotype independent of the direct cleavage of mRNA at UU↓U sequences by MazF-mt9. Instead, we tested whether the presence of RNase J now enabled 5′ RNA-seq detection of stalled ribosomes at AAA Lys codons in mRNAs just as it had in *M. tuberculosis*. Comparison of 5′ RNA-seq datasets of *E. coli* cells expressing MazF-mt9 ± RNase J revealed that ribosome stalling only occurred when RNase J was present (as indicated by the stacked AAA codon appearing on the kplogo at a distance consistent with ribosome stalling; Fig. 5a, b). This result suggested that RNase J collaborates with MazF-mt9 in *M. tuberculosis* cells, i.e., the toxin first inactivates a single tRNA (tRNA^Lys43-UUU^) and RNase J subsequently recognizes and cleaves mRNA on the 5′ side of ribosomes stalled at AAA Lys codons.

We next deleted the RNase J gene in *M. smegmatis* (MSMEG_2685) to more rigorously test the role of RNase J in mycobacteria. In contrast to the gain-of-function phenotype we observed upon coexpression of Rv2752 with MazF-mt9 in *E. coli*, we were surprised to find that there was no loss-of-function without MSMEG_2685 because we could still detect ribosome stalling by 5′ RNA-seq (Supplementary Fig. 3). Given the clear result in *E. coli*, both *M. smegmatis* and *M. tuberculosis* genomes might encode an additional functionally related enzyme with RNase J-like activity that complements the enzymatic activity we deleted. There is precedent for this type of functional redundancy as *Bacillus subtilis* has two related RNase J genes^25–27^. However, in contrast to *B. subtilis*, the other *M. tuberculosis* enzyme(s) that compensates for loss of RNase J might elude identification because it (they) may lack significant sequence similarity to RNase J. MazF-mt9 itself serves as precedent for functional similarity without sequence similarity. It is the only known tRNA-cleaving MazF family member in bacteria^15,16^. Its substrate specificity and cleavage activity mimics many tRNA-cleaving VapC toxins even though MazF-mt9 lacks sequence or structural similarity to any VapC family member^15,16^.

### Cleavage at stalled ribosomes favors AAA-deficient mRNAs

**Fig. 6:**
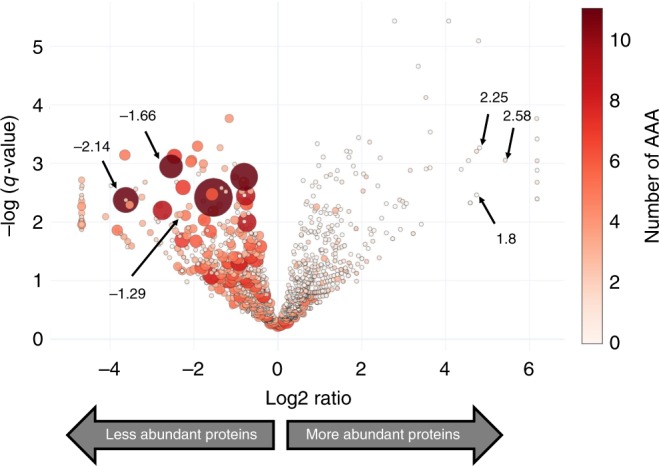
Induction of MazF-mt9 leads to global proteomic shifts based on the AAA codon content of transcripts. Volcano plot showing the proteomic changes in newly synthesized proteins between induced (+MazF-mt9) and uninduced cells. The diameter and color intensity of each represented protein (circle) is proportional to the number of AAA codons in its coding sequence. Fold changes in mRNA levels obtained by RNA-seq are shown for three examples of upregulated and downregulated proteins (arrows). The source data with full list of detected proteins is presented in Supplementary Data 2

The single cleavage site before one or more stalled ribosomes at AAA Lys codons is expected to result in a truncated, nonfunctional mRNA. This, in turn, should result in a decrease in the abundance of the proteins encoded by these transcripts. To test this directly, we metabolically labeled *M. smegmatis* cells with the methionine (Met) mimetic azidohomoalanine (AHA) whose azido group can be coupled to an alkyne-containing reagent using click chemistry^28^. AHA is efficiently incorporated into nascent proteins without cytotoxicity with a sensitivity comparable to [^35^S]Met labeling^29^. We selectively captured the newly synthesized proteins after MazF-mt9 was induced using an alkyne-containing column and performed an in-column trypsin digestion. The peptides released from the column were subjected to quantitative mass spectrometry. The results are graphed as a volcano plot where all individual proteins are represented as colored circles whose diameter and saturation is scaled to reflect AAA Lys codon content (Fig. 6, Supplementary Data 2). The effect of AAA Lys content on the abundance of newly synthesized protein is striking, with a clear downward shift in synthesis of AAA Lys codon-containing proteins relative to the uninduced control. These data also clearly illustrate that MazF-mt9 does not inhibit protein synthesis globally as there are hundreds of proteins that are synthesized in the presence of MazF-mt9.

**Fig. 7:**
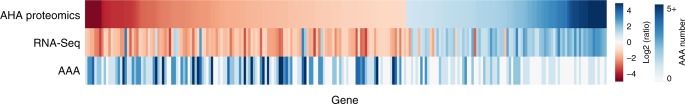
mRNA levels correlate with protein expression. Heatmaps comparing fold changes in newly synthesized proteins (AHA-labeling Proteomics, top row) and mRNA levels (RNA-Seq, middle row) in ±MazF-mt9 cultures. Genes with at least a log2ratio of ±1 were considered. The number of AAA codons (bottom row) of each gene is shown. Source data are provided as a tab within the Source Data file

We also pointed out three upregulated and downregulated proteins on each side of the volcano plot and included the respective fold-changes of their mRNAs obtained by conventional RNA-seq (see arrows and corresponding +/− values in Fig. 6). We extended the comparison of the three relevant parameters uncovered in this study—AAA content, fold-changes in proteins, and fold-changes in mRNAs—to a genome scale (Fig. 7). Comparison of these three analyses demonstrated that the anticipated trends were maintained genome-wide.

Finally, we demonstrated sustained protein synthesis after MazF-mt9 induction in *M. tuberculosis*. After metabolically labeling with AHA, newly synthesized AHA-containing *M. tuberculosis* proteins were coupled to the fluorophore TAMRA by click chemistry and visualized after PAGE (Supplementary Fig. 4). Comparison of ±MazF-mt9 samples revealed similar protein levels and band patterns (Supplementary Fig. 4).

### MazF-mt9 translation inhibition is codon specific

**Fig. 8:**
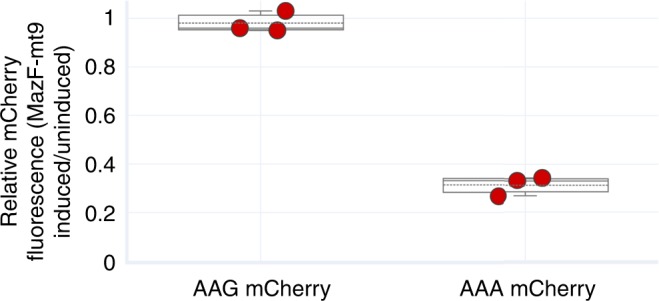
MazF-mt9 translation inhibition is codon specific. MazF-mt9 was co-expressed with two different versions of mCherry: one containing its lysine codons mutated to AAA (AAA mCherry) and the other to AAG (AAG mCherry). mCherry fluorescence was measured 5 h after induction of MazF-mt9 and normalized against an uninduced (−MazF-mt9) control. Each individual data point is derived from a biological replicate (*n* = 3). Box plot center line, median; box limits, upper and lower quartiles; whiskers, max and min values

To further solidify that MazF-mt9 expression leads to AAA Lys codon-directed inhibition of protein synthesis, we compared the level of protein synthesis in two transcripts engineered to contain only AAA Lys codons or AAG Lys codon. We used the mCherry fluorescence reporter gene as a convenient vehicle for translation read-out. Consistent with the single tRNA^Lys43-UUU^ target and the series of downstream events documented above, we measured a 70% reduction in mCherry fluorescence in the AAA-containing gene relative to the AAG only counterpart (Fig. 8).

## Discussion

**Fig. 9:**
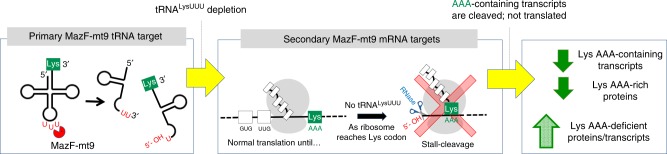
MazF-mt9 mechanism of action. The toxin controls gene expression through the cleavage of tRNA^Lys43-UUU^ (left panel), causing ribosome stalling and subsequent degradation of the mRNAs by one or more RNases (middle panel). This cooperative action reduces the steady levels of AAA-rich proteins and favors an increase in AAA-deficient proteins (right panel)

In summary, 5′ RNA-seq captured the unexpected, highly precise “one-two punch” dealt by the MazF-mt9 toxin that would typically require parallel Ribo-seq and RNA-seq analyses. MazF-mt9 inactivated just one tRNA, tRNA^Lys43-UUU^ in *M. tuberculosis* cells through UU↓U cleavage at its anticodon. Concordant with the depletion of tRNA^Lys43-UUU^, the dataset revealed compelling evidence of ribosome stalling exclusively at cognate AAA Lys codons (rare in the 65.6% GC-rich *M. tuberculosis* genome). These stalled ribosome signatures were detected in the 5′ RNA-seq dataset because another RNase—possibly RNase J working in concert with one or more addition RNases—cleaved the transcript 5′ of the stalled ribosomes. We confirmed that this RNase cleavage of AAA Lys codon-containing mRNA transcripts is associated with their removal from the transcriptome since the abundance of proteins encoded by AAA-containing mRNAs decreased in cells expressing MazF-mt9. Thus, we have tracked a succession of unexpected events set in motion by inactivation of only one tRNA in *M. tuberculosis* cells, which revealed an intricate mechanism for precise transcriptome recalibration that in turn reprograms the *M. tuberculosis* proteome (summarized in Fig. 9).

This cascade of molecular events is initiated by a single, highly specific, cleavage event at the ^35^UU↓U^37^ site within the anticodon of tRNA^Lys43-UUU^. MazF-mt9 specifically cleaves the tRNA^Lys43-UUU^, no other tRNA, not even the other Lys tRNA isoacceptor tRNA^Lys19-CUU^. Wobble rules dictate that tRNA^Lys43-UUU^ can recognize the less common Lys AAA codon (5.3/1000 codons), as well as the more frequently represented Lys codon in *M. tuberculosis*, AAG (15/1000 codons). However, MazF-mt9 cuts and inactivates only tRNA^Lys43-UUU^, resulting in a short supply of the tRNA that services both AAA and AAG Lys codons. For tRNA^Lys19-CUU^, wobble rules only allow recognition to Lys AAG codons. Since we did not detect stalling at AAG codons upon expression of MazF-mt9, tRNA^Lys19-CUU^ must be able to adequately supply Lys amino acids to AAG codons in growing peptide chains.

Paradoxically, although TA toxins are unusually abundant in *M. tuberculosis*, they have been proposed to universally act by simply inhibiting protein translation^30^. However, our data does not support this model. MazF-mt9 did not inhibit translation in *M. tuberculosis* (Supplementary Fig. 4). In fact, there are hundreds of newly synthesized proteins in our quantitative mass spectrometry dataset (Fig. 6). Instead, MazF-mt9 appears to reprogram *M. tuberculosis* physiology and growth by enlisting codon-bias as a means to surgically remove distinct subsets of mRNA transcripts. This targeted transcript ablation leads to the global reduction in the levels of the proteins they encode, and likely enables facile adaptation to stress. It appears that the growth arrest phenotype associated with MazF-mt9-mediated proteome remodeling might be a consequence of the calibrated increase or reduction of key regulatory proteins or enzymatic pathways that influence growth rate based on their codon content.

Finally, our findings are consistent with codon-biased translation reported by the Dedon laboratory upon hypoxic persistence in *M. bovis*^31^. However, in their case the codon-biased translation was attributed to alterations in tRNA modification tracked by mass spectrometry, not tRNA abundance^31^. The MazF-mt9 toxin-mediated, codon-driven shift in the transcriptome is predicted to alter the spectrum of proteins in *M. tuberculosis* cells and sabotage the efficacy of the immunological and therapeutic assaults intended to eradicate this deadly pathogen. Since there are many predicted tRNA-cleaving toxins in *M. tuberculosis*, the activity of MazF-mt9 might be the first of several examples that enlist this approach to selectively reprogram cell physiology. However, the mechanics of the putative orchestrated enlistment of multiple tRNA-cleaving toxins are unclear since physiological triggers have not yet been identified for any of these toxins in *M. tuberculosis*.

## Methods

### Strains, plasmids, and reagents

All experiments were performed using either *M. tuberculosis* strain H37Rv (ATCC 25618), *M*. *tuberculosis* mc^2^ 6206 (*ΔpanCD ΔleuCD*, generously provided by William Jacobs laboratory, Albert Einstein College of Medicine), *M. smegmati*s mc^2^ 155 (ATCC 700084), or *Escherichia coli* BW25113Δ6. *E. coli* BW25113Δ6 (a gift from the Masayori Inouye laboratory, Rutgers RWJMS) is a derivative of BW25113^32^ that lacks loci for six *E. coli* chromosomal TA modules (*mazEF*, *chpBIK*, *relBE*, *hipBA*, *yefM-yoeB*, and *dinJ-yafQ*). For experiments using mycobacteria, MazF-mt9 (Rv2063A locus) was amplified by PCR from genomic DNA extracted from *M*. *tuberculosis* H37Rv, using oligonucleotides containing ClaI and EcoRV restriction sites at the 5′ and 3′ ends respectively (NWO1646—5′ CCCATCGATGGCTGAGCCACGGCGAG 3′ and NWO1647—5′ CGATATCTCACGGGTCCCGGCCACC 3′). The resulting fragment was cloned adjacent to an anhydrotetracycline (ATC)-inducible promoter in the pMC1s plasmid^33^. MazF-mt9 expression was induced by adding ATC (Clontech) to the media to a final concentration of 200 ng/ml.

*M. tuberculosis* H37Rv and *M. smegmatis* mc^2^ 155 cells were grown in 7H9 Middlebrook medium with a final concentration of 0.05% Tween 80, 0.5% bovine albumin, 0.2% dextrose, 0.085% NaCl (7H9-TW80-ADN) and 20 μg/ml kanamycin or zeocin a 25 μg/ml. The attenuated strain mc^2^ 6206 was grown in 7H9 Middlebrook media containing 1× OADC supplement (Sigma), 0.05% of tyloxapol, 50 µg/ml of pantothenic acid, 100 µg/ml of leucine, and kanamycin at 20 μg/ml. For co-expression experiments in *E. coli*, MazF-mt9 was cloned into the arabinose-inducible vector pBAD33^32^ and *M. tuberculosis* RNase J (Rv2752c) was cloned into the IPTG-inducible pinIII plasmid. *E. coli* harboring *mazF-mt9*-pBAD33 and/or RNase J-pinIII was grown in M9 minimal media using glycerol as carbon source and chloramphenicol at 25 μg/ml or ampicillin at 100 μg/ml as selection markers. The inducers (arabinose and/or IPTG) were added to the media at final concentration of 0.2% and 1 mM, respectively. All growth experiments were conducted at 37 °C and under constant agitation at 230 rpm.

For co-expression experiments in *M. smegmatis*, codon-adjusted mCherry was cloned into the nitrile-inducible plasmid pNIT-zeo^34^. *M. smegmatis* containing both pMC1s-mazF-mt9 and pNIT-zeo-mCherry were grown in triplicate to OD_600nm_ 0.2 and co-induced for 5 h by adding ATC and isovaleronitrile to a final concentration of 200 ng/ml and 1 μM, respectively. mCherry fluorescence (Ex: 585 nm/Em: 610 nm) and ODs were measured on a Synergy HT spectrophotometer (Biotek) and normalized against a -ATC (no MazF-mt9 induction) control group. Background fluorescence signal was inferred by measuring mCherry uninduced cultures and subtracted from all other groups.

### RNA isolation

To isolate RNA from cells expressing MazF-mt9, bacteria were grown in the absence or presence of inducer for 2 or 7 days (corresponding growth curve included as Supplementary Fig. 2) for *M. tuberculosis*, 7.5 h for *M. smegmatis* or 60 min for *E. coli*. ATC was replenished into the media every 48 h, as necessary.

Cells were centrifuged at 2000 × *g* for 10 min at 4 °C, and supernatants were removed. Mycobacterial cell pellets were washed three times with PBS, resuspended in 1 ml of Trizol and approximately 200 µl of 0.1 mm zirconia or glass beads were added to the tube. Cells were lysed in 4 cycles of 30 s at 9000 rpm using Precellys Evolution homogenizer (Bertin Corp.) or 4 times at 7000 rpm for 45 s using a MagNA Lyser (Roche) with 1 min intervals on ice in between each cycle. The lysate was centrifuged at 12,000 rpm at 4 °C and total RNA was extracted from the supernatant using Direct-zol™ RNA MiniPrep Plus (Zymo Research). RNA was eluted in nuclease-free water, treated with TURBO DNase (Thermo Fisher) for 30 min at 37 °C, and re-purified using the same RNA extraction kit. The extracted RNA was quantified by spectrophotometry using a µCuvette in a BioSpectrometer (Eppendorf) or Nanodrop (Thermo Fisher).

### Northern analysis of tRNA levels

To detect mycobacterial tRNA, specific DNA probes complementary to the 3′ end of *M. tuberculosis* tRNA^Lys43-UUU^, tRNA^Lys19-CUU^ and tRNA^Pro14-GGG^ genes (NWO2517 5′-TGCCCCCACCAGGACTCGAACCTGGGAC-3′, NWO2570 5′-GCCGTCAGGGTTTCGAACCCC-3′ and NWO2518 5′-GTCAAGTGGTCGCAGGTTCAAATCCTGTCAGC-3′, respectively) were radiolabeled at the 5′ end with T4 polynucleotide kinase (New England Biolabs) and [γ-^32^P]ATP (PerkinElmer) for 1 h at 37 °C. Then, total RNA (2 µg) from *M. tuberculosis* was resolved on a 9% polyacrylamide, 7 M urea gel and stained with SYBR Gold (Invitrogen) to assess and ensure overall quality. The RNA was transferred to a nylon Hybond-N+ membrane (GE Healthcare) and hybridized with the ^32^P-labeled oligonucleotides at 45 °C overnight. To further remove non-specific signals, the membrane was washed twice with 0.01% SDS and 0.1× SCC at 50 °C for 10 min. The membranes were exposed to phosphorimager screens and scanned using the Typhoon FLA 9500 (GE Healthcare) image system.

### Labeling of newly synthesized *M. tuberculosis* proteins

To assess the levels of newly synthesized proteins in *M. tuberculosis* mc^2^ 6206 after toxin induction, MazF-mt9 was induced for 72 h and cultures (uninduced and induced) were incubated with AHA for 5 h, in duplicate. Cells were then pelleted and lysed in 4 cycles of 30 s at 9000 rpm using Precellys Evolution homogenizer (Bertin). The lysate was centrifuged, and the supernatant used as input in the Click-IT Protein Reaction Buffer kit (ThermoFisher), linking the AHA-containing proteins with an alkyne-containing version of the fluorophore TAMRA (ThermoFisher). Twenty microgram of protein for each sample were run on a SDS-PAGE gel and subsequently scanned using the Typhoon FLA 9500 (GE Healthcare) image system.

### 5′ RNA-seq

Preparation of 5′-dependent libraries was performed as described in Schifano et al.^20^. Briefly, for sequencing of RNAs containing 5′ hydroxyl ends (5′-OH), total RNA from pMC1s-mazF-mt9 (+ATC and −ATC) samples was digested using 1 U of Terminator 5′-Phosphate-Dependent Exonuclease (Epicenter) to remove RNAs containing 5′-monophosphate (5′-P). Subsequently, the RNA was purified using RNA Clean & Concentrator™-5 (Zymo Research) and 5′-OH ends were phosphorylated using 3 U of T4 PNK (New England Biolabs) to allow subsequent adapter ligation. The Illumina small RNA 5′ adapter (5′-GUUCAGAGUUCUACAGUCCGACGAUCNNNNNN-3′) was ligated using T4 RNA ligase 1 at 16 °C overnight (New England Biolabs), according to the manufacturer’s instructions. Excessive unbound adapters were removed by gel excision and purification. The purified RNA was used in a Superscript IV reverse transcription reaction (ThermoFisher) using the primer 5′-GCCTTGGCACCCGAGAATTCCANNNNNNNNN-3′ and the resulting cDNA was gel extracted, selecting fragments from 80 to 500 nts. The cDNA libraries were amplified in 12 cycles of PCR using Phusion HF DNA polymerase (ThermoFisher). The oligonucleotides used for PCR amplification were RP1 (5′- AATGATACGGCGACCACCGAGATCTACACGTTCAGAGTTCTACAGTCCGA -3′) and RPIX (5′-CAAGCAGAAGACGGCATACGAGATNNNNNNGTGACTGGAGTTCCTTGGCACCCGAGAATTCCA-3′), where the *N*’s represent the individual Illumina barcodes for each library. After electrophoresis on a 10% (wt/vol) polyacrylamide gel, amplified DNA between the sizes 150 and 450 bp was isolated by gel excision and sequenced in an Illumina NextSeq 500 or HiSeq 2500 platform.

The FASTQ files generated by the 5′ RNA-seq protocol had the adapter and the first 6 nucleotides of the high quality end trimmed using Trimmomatic^35^. Then, the sequences were trimmed to 20 nts (discarding shorter sequences) and aligned either to *M. tuberculosis* genome (Genbank accession: AL123456), *M. smegmatis* (Genbank accession: CP000480) or to *E. coli* (Genbank accession: NC_000913) using Bowtie 1.2.0 applying the parameters –n 0 –l 20^36^. For each nucleotide in the genome, we calculated the number of reads that started at that position (i.e. the amount of RNA molecules that had their 5′ end starting at that nucleotide in the genome). Read counts were normalized to sequencing depth and expressed as “reads per million of mapped reads” (rpm). Next, we calculated the ratio for each position (counts in induced sample/counts in uninduced sample) to generate a fold change. Positions that had 0 counts in the uninduced library were adjusted to a pseudo-count of 1. We only considered reads that had at least 5 rpm for mRNAs and 50 rpm for tRNAs in the induced sample and a ratio of fold change of at least 10. For nucleotide frequency visualization, we ran weblogo^37^ on the top hits using the default parameters. kpLogo^38^ was used adjusting to 3-mers to preferentially find codons in the experiments using RNase J and MazF-mt9 co-expressing *E. coli*; the Bonferroni corrected p-value was used to find significant k-mer positions. The significant hits from the 5′ RNA-seq from a culture expressing RNase J only (compared to an uninduced culture) was used as background hits in the KpLogo program in order to remove RNase J cleavage products in the absence of MazF-mt9.

### RNA-seq

In order to deplete 16S/23S ribosomal RNA from the extracted RNA, the samples were treated using the RiboZero (Illumina) kit for bacteria. Approximately 100 ng of rRNA-depleted RNA were used to generate the libraries using NEBNext Ultra II Directional RNA Library Prep Kit for Illumina (New England Biolabs) and sequenced on an Illumina HiSeq 2500 or similar. The resulting sequences were mapped to the *M. smegmatis* reference genome (Genbank accession: CP000480) using the default parameters of Bowtie 1.2. Stringtie 1.3.4^39^ and DESeq2 2.11.40.2^40^ programs were used for transcript assembly and differential expression analysis, respectively.

### Ribo-seq

In order to confirm ribosome stalling, we analyzed the position of translating ribosomes on the mRNA through Ribo-Seq^41,42^. *M. smegmatis* or *M. tuberculosis* cells were grown to OD600nm = 0.2, separated into induced (+ATC) and uninduced (−ATC) cultures and incubated at 37 °C for 7.5 h (*M. smegmatis*) or 72 h (*M. tuberculosis*). The cells were harvested by quick filtration using a 0.22 μm filter apparatus. Immediately after, cells were quickly scraped off the filter surface using a scoopula and flash frozen in liquid nitrogen. Cells were ruptured while frozen through bead beating with the Precellys Evolution (Bertin Corp). The procedure was performed at 4 °C using 7-ml metal tubes and metal beads for 8 cycles of 30 s at 6000 rpm. Cells were kept in liquid nitrogen for 30 s between each cycle. After lysis, the resulting cell powder was resuspended in lysis buffer (step 7A, Becker et al.^41^), and the Becker et al. no-crosslinking protocol was followed^41^. The A-site positioning was predicted using RiboTools^43^ and the coverage around specific codons was analyzed using bamCoverage of the DeepTools^44^ package.

### Construction of RNase J deletion strain

An RNase J mutant strain (MSMEG_2685) was constructed using the ORBIT recombineering method^22^. We designed the Bxb1 attP-containing oligonucleotide with the first and last 70 base pairs of the MSMEG_2685 gene (NWO3007, 5′-TCAGATCTCTATGACGGTCGGGACGATCATCGGCTGCCTGCGGTAGGTCTCGCCCACCCACTTGCCGACCGGTTTGTACCGTACACCACTGAGACCGCGGTGGTTGACCAGACAAACCTACCGCCCAGGGCGGTGACACGCAGTCCGCCCGGAGCGAGCGGTGGTGGCGGCGCGAGTTCGGCGCTCAT -3′). To confirm successful deletion, we performed PCR using oligonucleotides NWO3008 (5′-GGG GAT TCA TGA GCG CCG A-3′) and NWO3009 (5′-ACG CGT ATG TCG CGT TGG AG-3′), which are complimentary to the flanking regions of the deleted gene, as well as whole genome sequencing.

### Proteomics

To assess newly synthesized proteins, *M. smegmatis* cultures (induced or uninduced, in triplicate) were incubated for 2 h with azidohomoalanine (AHA, ThermoFisher). AHA is an azide-containing Met analog that is incorporated into proteins, allowing the capture of the newly synthesized, AHA-incorporated proteins by click chemistry. We selectively captured the newly synthesized proteins using an alkyne-containing column from the Click-iT™ Protein Enrichment Kit (ThermoFisher), following the manufacturer's protocol, and proceeded with in-column trypsin digestion.

Digests were analyzed using a Q Exactive HF tandem mass spectrometer coupled to a Dionex Ultimate 3000 RLSCnano System (Thermo Scientific). Samples were solubilized in 5% acetonitrile/0.1% TFA and loaded onto a fused silica trap column Acclaim PepMap 100, 75 µm × 2 cm (ThermoFisher). After washing for 5 min at 5 µl/min with solvent A (0.2% formic acid), the trap column was brought in-line with an analytical column (Nanoease MZ peptide BEH C18, 130 A, 1.7 µm, 75 µm × 250 mm, Waters) for LC-MS/MS. Peptides were eluted using a segmented linear gradient from 4 to 90% B (B: 0.08% formic acid, 80% acetonitrile): 4% B for 5 min, 4–15% B for 30 min, 15–25% B for 40 min, 25–50% B for 44 min and 50–90% B for 8 min. Mass spectrometry data was acquired using a data-dependent acquisition procedure with a cyclic series of a full scan with resolution of 120,000 followed by MS/MS (HCD, relative collision energy 27%) of the 20 most intense ions and a dynamic exclusion duration of 20 s.

The raw LC-MS data was converted into MASCOT Generic Format (MGF) using Proteome Discover 2.1 (ThermoFisher) and searched against the NCBI *M. smegmatis* database (Accession: CP000480) together with a database of common laboratory contaminants (http://www.thegpm.org/crap/) using a local implementation of the global proteome machine (GPM Fury)^45^.

Differential expression was estimated using the QLSpline option of the QuasiSeq package, only using proteins having 10 or more spectral counts total (from the 6 LC-MS/MS runs on the six different samples (https://cran.r-project.org/web/packages/QuasiSeq/index.html)^46^. Data is presented as estimated log_2_ ratios of induced/uninduced. *Q*-values are calculated using the fdrtool package of Strimmer^47^ with significant changes at or below a *q*-value of 0.05.

### Reporting summary

Further information on research design is available in the Nature Research Reporting Summary linked to this article.

## Supplementary information

Supplementary Information

Description of Additional Supplementary Files

Supplementary Data 1

Supplementary Data 2

Reporting Summary

Source Data

## Data Availability

The sequencing datasets generated in this study were deposited in the NCBI Sequence Read Archive under BioProject accession number PRJNA490371. Mass spectrometry data were deposited in the MassIVE database under the accession number MSV000083670. The source data underlying Figs. 1c, 3d, h, 6, and 7, and Supplementary Fig. 4 are provided as a Source Data file and Supplementary Data 2. A reporting summary for this Article is available as a Supplementary Information file.
