# Supplementary material for: Toxin-mediated ribosome stalling reprograms the Mycobacterium tuberculosis proteome
Source: Nat Commun. 2019 Jul 10;10:3035. doi: 10.1038/s41467-019-10869-8 (PMC6620280; doi:10.1038/s41467-019-10869-8)
Supplement: Supplementary file 1 — Supplementary Information [file 41467_2019_10869_MOESM1_ESM.pdf]

SUPPLEMENTARY INFORMATION

Toxin-mediated ribosome stalling reprograms the *Mycobacterium tuberculosis* proteome

Barth et al.

a

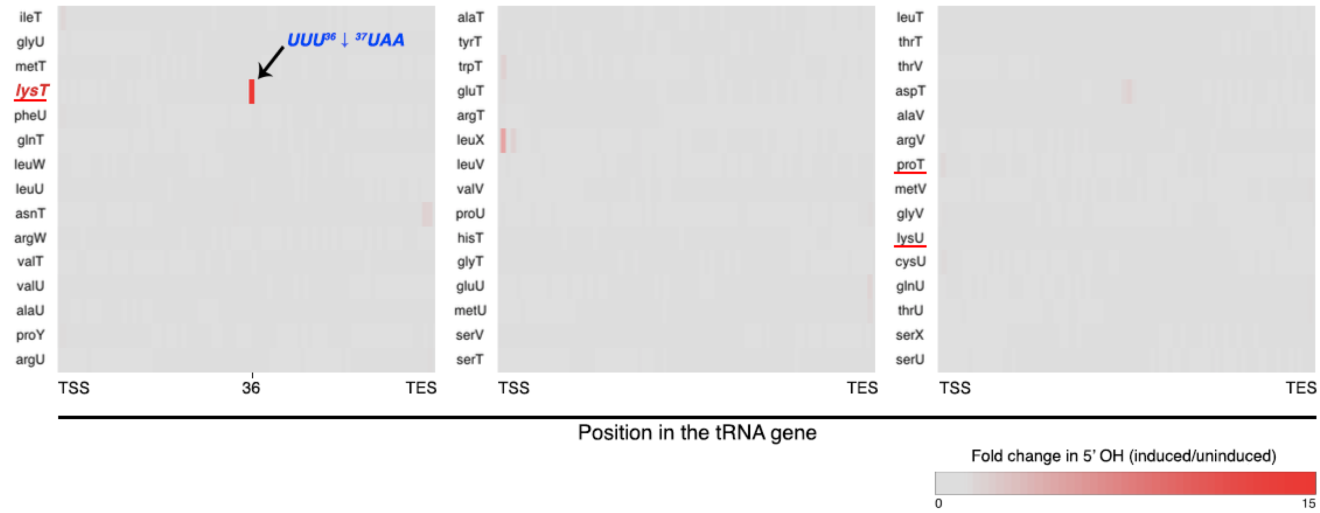

b

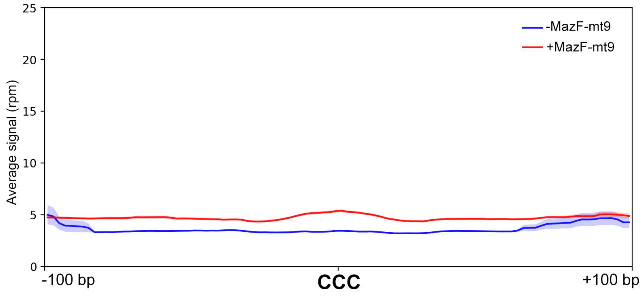

c

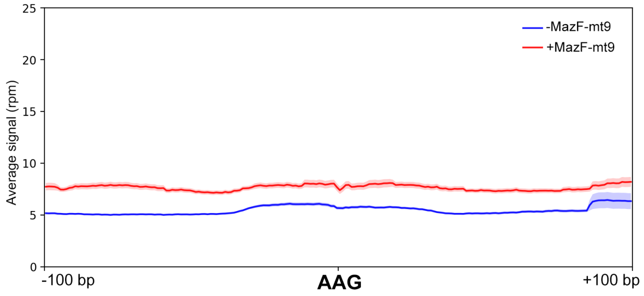

Supplementary Fig. 1. Only tRNA$^{Lys43\text{-}UUU}$ is cleaved by MazF-mt9 in *M. tuberculosis*. a) Heat map representing fold changes in tRNA cleavage of all 45 tRNA species when MazF-mt9 is expressed for 2 days in H37Rv *M. tuberculosis* cells relative to the uninduced control. The previously identified in vitro targets *proT* and *lysU*, which were not confirmed in vivo, are underlined in red. TSS, transcription start site; TES, transcription end site. Cleavage of tRNA$^{Lys43\text{-}UUU}$ is shown at position 36 within the anticodon. b-c, Average read counts (reads per million, rpm) of mapped ribosome footprints surrounding the cognate codons of the in vitro mistargets *proT* tRNA (b) or the other Lys isoacceptor tRNA *lysU* (c) in *M. tuberculosis*, showing no significant stalling. Blue or red solid line represent uninduced cells or cells expressing MazF-mt9, respectively, with the light-colored shading denoting standard deviation.

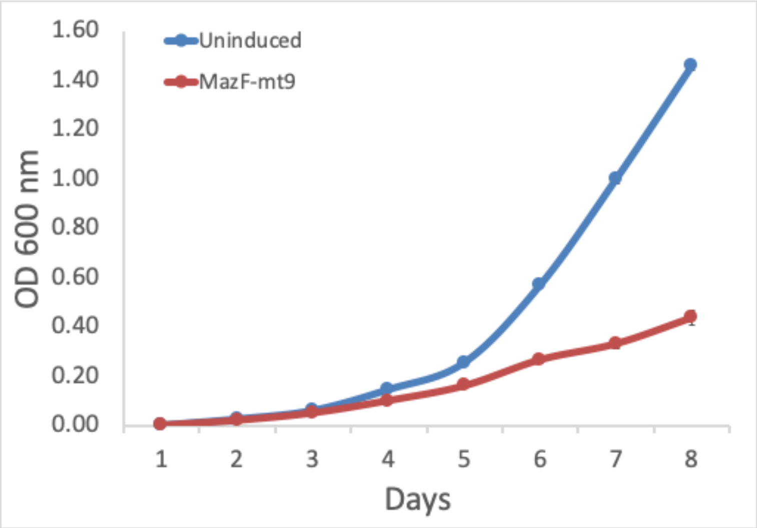

Supplementary Fig. 2. MazF-mt9 expression leads to slow growth in *M. tuberculosis*. *M. tuberculosis* cultures harboring pMC1s-MazF-mt9 were grown in 7H9 media in the presence (red) or absence (blue) of inducer (ATC) for 8 days. To estimate growth, absorbance at 600 nm was measured every 24 h. Error bars represent the standard error of the mean (S.E.M.) obtained from three biological replicates.

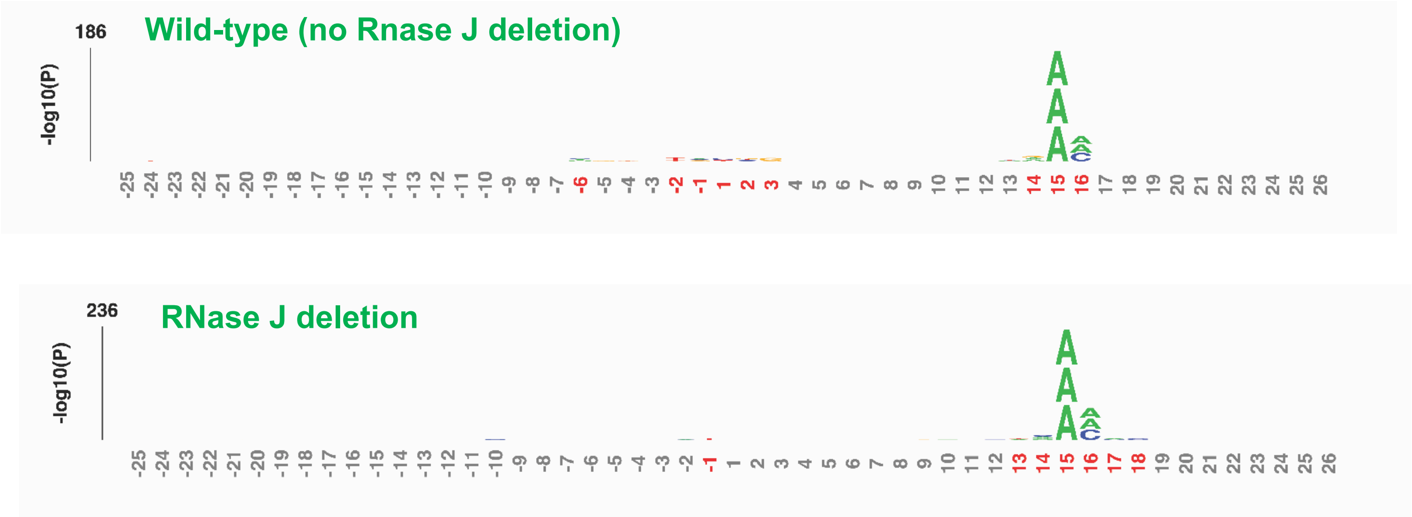

Supplementary Fig. 3. Ribosome stalling occurs upon expression of MazF-mt9 in a RNase J-deletion background. An *M. smegmatis* mutant lacking the entire RNase J gene (MSMEG_2685) was analyzed with 5' RNA-seq after expressing MazF-mt9 for 5 h and compared to an uninduced mutant sample. Positions are numbered related to the cleavage site. Enrichment of Lys AAA codon is still seen at position +15, suggesting the existence of a functionally compensatory RNase in mycobacteria. The wild-type (no MSMEG_2685 RNase J deletion) kplogo shows essentially the same pattern of stalling with the stacked AAA sequence ~15 nts from the cleavage site.

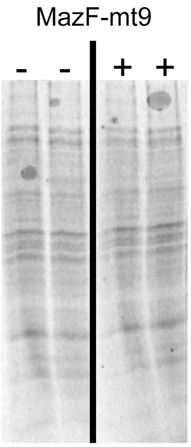

Supplementary Fig. 4. MazF-mt9 expression does not inhibit translation in *M. tuberculosis*. Newly synthesized proteins from two biological replicates of *M. tuberculosis* cultures with (+) or without (-) MazF-mt9 expression were visualized by fluorescent imaging after metabolic labeling with AHA, click-chemistry coupling to the fluorophore TAMRA, followed by SDS-PAGE.
