# Supplementary material for: Toxin-mediated ribosome stalling reprograms the Mycobacterium tuberculosis proteome
Source: Nat Commun. 2019 Jul 10;10:3035. doi: 10.1038/s41467-019-10869-8 (PMC6620280; doi:10.1038/s41467-019-10869-8)
Supplement: Supplementary file 2 — Description of Additional Supplementary Files [file 41467_2019_10869_MOESM2_ESM.pdf]

Description of Additional Supplementary Files

File Name: Supplementary Data 1
Description: 5’ RNA-seq mRNA dataset: MazF-mt9 induced for 7 days in H37Rv *M. tuberculosis* cells. Cleavage is observed immediately before the nucleotide indicated by a capital letter. The flanking DNA sequences were added 25 nt up- and downstream of the cleavage site.

File Name: Supplementary Data 2
Description: Mass spectrometry data of the newly synthesized proteins in *M. smegmatis* expressing MazF-mt9 normalized to uninduced cultures.
