# Supplementary material for: Toxin-mediated ribosome stalling reprograms the Mycobacterium tuberculosis proteome
Source: Nat Commun. 2019 Jul 10;10:3035. doi: 10.1038/s41467-019-10869-8 (PMC6620280; doi:10.1038/s41467-019-10869-8)
Supplement: Supplementary file 5 — Reporting Summary [file 41467_2019_10869_MOESM5_ESM.pdf]

nature research

Corresponding author(s): Nancy A. Woychik

Last updated by author(s): May 30, 2019

Reporting Summary

Nature Research wishes to improve the reproducibility of the work that we publish. This form provides structure for consistency and transparency in reporting. For further information on Nature Research policies, see Authors & Referees and the Editorial Policy Checklist.

Statistics

For all statistical analyses, confirm that the following items are present in the figure legend, table legend, main text, or Methods section.

n/a	Confirmed	
☐	☒	The exact sample size (*n*) for each experimental group/condition, given as a discrete number and unit of measurement
☐	☒	A statement on whether measurements were taken from distinct samples or whether the same sample was measured repeatedly
☐	☒	The statistical test(s) used AND whether they are one- or two-sided<br>*Only common tests should be described solely by name; describe more complex techniques in the Methods section.*
☒	☐	A description of all covariates tested
☐	☒	A description of any assumptions or corrections, such as tests of normality and adjustment for multiple comparisons
☐	☒	A full description of the statistical parameters including central tendency (e.g. means) or other basic estimates (e.g. regression coefficient) AND variation (e.g. standard deviation) or associated estimates of uncertainty (e.g. confidence intervals)
☐	☒	For null hypothesis testing, the test statistic (e.g. *F*, *t*, *r*) with confidence intervals, effect sizes, degrees of freedom and *P* value noted<br>*Give P values as exact values whenever suitable.*
☒	☐	For Bayesian analysis, information on the choice of priors and Markov chain Monte Carlo settings
☒	☐	For hierarchical and complex designs, identification of the appropriate level for tests and full reporting of outcomes
☒	☐	Estimates of effect sizes (e.g. Cohen's *d*, Pearson's *r*), indicating how they were calculated

Our web collection on statistics for biologists contains articles on many of the points above.

Software and code

Policy information about availability of computer code

Data collection	no data collection software was used.
Data analysis	For data analysis, the following software were used: Trimmomatic 0.36.5, Bowtie 1.2, weblogo 3.6, kpLogo web version (2017), RiboTools, Deeptools 3.1.3, QuasiSeq 1.0-10-2, StringTie 1.3.4 and DESeq 2.11.40.2.

For manuscripts utilizing custom algorithms or software that are central to the research but not yet described in published literature, software must be made available to editors/reviewers. We strongly encourage code deposition in a community repository (e.g. GitHub). See the Nature Research guidelines for submitting code & software for further information.

Data

Policy information about availability of data

All manuscripts must include a data availability statement. This statement should provide the following information, where applicable:

- Accession codes, unique identifiers, or web links for publicly available datasets
- A list of figures that have associated raw data
- A description of any restrictions on data availability

The sequencing datasets generated in this study were deposited in the NCBI Sequence Read Archive under BioProject accession number “PRJNA490371 [https://www.ncbi.nlm.nih.gov/Traces/study/?acc=PRJNA490371]”. Mass spectrometry data was deposit in the MassIVE database under the accession number “MSV000083670[ftp://massive.ucsd.edu/MSV000083074]”. The source data underlying Fig. 1c, Fig. 3 panels d and h, Fig. 6 and 7, and Supplementary Figure 4 are provided as a Source Data file and Supplementary Data 2. A reporting summary for this Article is available as a Supplementary Information file.

Field-specific reporting

Please select the one below that is the best fit for your research. If you are not sure, read the appropriate sections before making your selection.

☒ Life sciences ☐ Behavioural & social sciences ☐ Ecological, evolutionary & environmental sciences

For a reference copy of the document with all sections, see nature.com/documents/nr-reporting-summary-flat.pdf

Life sciences study design

All studies must disclose on these points even when the disclosure is negative.

Sample size	For most experiments, we used triplicates which is widely accepted in the field of molecular biology.
Data exclusions	Due to the by the high sensitivity of next-generation sequencing techniques, we applied a cut-off of 5 rpm to mRNA hits and 50 rpm in tRNA hits for induced samples in our 5' RNA Seq method to eliminate weak signals and background noise.
Replication	We performed each experiment independently at least twice to ensure reproducibility of the reported results.
Randomization	No randomization was required in our studies, as our study organisms are derived from well-established reference strains.
Blinding	Blinding was not necessary for data acquisition in our study, due to the nature of our research. Biological samples are assigned alias names during handling to avoid bias in treating certain samples differently. For data analysis, our datasets are treated as library names which are reassigned into their original sample description at the end of the workflow.

Reporting for specific materials, systems and methods

We require information from authors about some types of materials, experimental systems and methods used in many studies. Here, indicate whether each material, system or method listed is relevant to your study. If you are not sure if a list item applies to your research, read the appropriate section before selecting a response.

Materials & experimental systems

n/a	Involved in the study	
☒	☐	Antibodies
☒	☐	Eukaryotic cell lines
☒	☐	Palaeontology
☒	☐	Animals and other organisms
☒	☐	Human research participants
☒	☐	Clinical data

Methods

n/a	Involved in the study	
☒	☐	ChIP-seq
☒	☐	Flow cytometry
☒	☐	MRI-based neuroimaging
